# Causal AI digital twin for bioprocess bottleneck diagnosis via metabolic flexibility and rigidification maps

**DOI:** 10.1016/j.isci.2026.116820

**Published:** 2026-07-16

**Authors:** Changman Kim, Hyeongwoo Choi, Dukwoo Kim

**Affiliations:** 1 Department of Biotechnology and Bioengineering, Chonnam National University, Gwangju 61188, Republic of Korea; 2 Department of Earth Systems and Environmental Sciences, Chonnam National University, Gwangju 61188, Republic of Korea

**Keywords:** digital twin, genome-scale metabolic model, flux variability analysis, metabolic flexibility, explainable AI, SHAP, causal discovery, regime shifts, bottleneck diagnosis, Stenotrophomonas maltophilia

## Abstract

Diagnosing underperforming non-model microbial cultures remains difficult. To address this, we developed an intervention-aware genome-informed digital twin with interpretable flexibility features, explainable learning, and causal-structure discovery to convert sparse anchors into actionable diagnostic artifacts. We grew *Stenotrophomonas maltophilia* SO-1 aerobically on acetate minimal medium as a controlled testbed and anchored the twin with standardized harvest-time growth phenotypes (OD_600_ at 32 h). We then generated an intervention-labeled design space via Latin hypercube sampling (LHS), labeled regimes using the active-constraint set, and encoded intracellular states utilizing targeted flux-variability analysis (FVA) widths across 30 reactions/modules. Explainable learning via XGBoost + SHapley Additive exPlanations (SHAP) identified regime-specific signatures and tipping-like patterns consistent with flexibility collapse, while causal-structure discovery yielded a rigidification map of bottleneck hypotheses into an upstream-to-downstream cascade. Validation under acetate-stress, nutrient-limited, and oxygen-transfer conditions yielded regime-level diagnostic agreement and highlighted systematic mismatches as signals for non-stoichiometric constraints.

## Highlights

• A genome-informed digital twin is anchored by 32 h OD600 readouts in a non-model bioprocess
• Design-space exploration (LHS, n = 2,000) reveals discontinuous regime boundaries
• SHAP flags flexibility collapse in FVA-width features as the main impairment
• Causal discovery yields a rigidification map prioritizing testable interventions

## Graphical abstract

**Figure:**
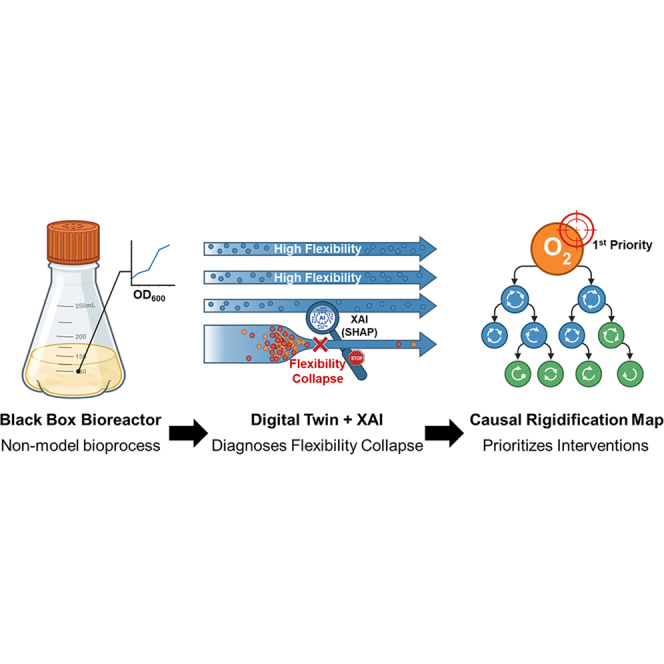

## Teaser

Genetics; Molecular biology; Immunology; Microbiology; Cell biology; Computational bioinformatics

## Introduction

Industrial bioprocesses inherently operate under complex and shifting conditions, facing challenges such as variable feedstocks, limited oxygen availability, and heterogeneous microenvironments.^1^^,^^2^ When performance deviates from the anticipated target, operators require more than simple status updates of the titer and/or optical density; they also need a diagnostic explanation that clarifies the underlying metabolic state.^3^ Specifically, they must be able to determine which metabolic regime the culture currently occupies, which constraint is functionally limiting, and which intervention is most likely to restore performance. Recent hybrid frameworks, such as COSMIC-dFBA, have successfully addressed this by using machine learning to predict metabolic state shifts in data-rich mammalian cell cultures.^3^ However, extending such diagnostics to non-model bioprocessing microorganisms remains challenging, particularly where curated pathway knowledge is scarce, and mapping of the external conditions to a particular internal failure mode remains uncertain.

While modern fermentation platforms generate data-rich environments, measurements that offer the most mechanistic insight (such as omics data) are rarely deliverable within the decision-relevant window required for real-time control.^4^^,^^5^^,^^6^ Consequently, despite the abundance of data, operational decisions are often driven by empirical heuristics rather than a deep understanding of the cellular state. To bridge this gap, genome-scale metabolic models (GEMs) have emerged as a promising foundation for providing digital twins that offer a mechanistic framework for linking external conditions to intracellular phenotypes through stoichiometric and thermodynamic constraints.^7^^,^^8^ However, translating GEM-based simulations into actionable diagnostics is fraught with challenges. The primary obstacle is the non-uniqueness of flux solutions. Because many different intracellular metabolic flux states can satisfy the same set of external constraints, point estimates of flux are often unstable and unreliable as diagnostic targets.^9^ Conversely, purely data-driven machine-learning approaches can map process data to outcomes with high accuracy, albeit often lacking mechanistic interpretability.^10^ Furthermore, these black-box models can behave unpredictably when the system is subjected to interventions that shift it outside the training distribution, which is precisely when troubleshooting is most needed.^10^

Herein, we introduce an intervention-aware diagnostic platform that integrates the mechanistic structure of a digital twin with the diagnostic power of explainable artificial intelligence (XAI) and causal discovery. The central conceptual shift in our approach is moving away from diagnosing a specific static flux state^11^ and instead focusing on diagnosing metabolic rigidification. The latter is defined as the loss of metabolic degrees of freedom, which indicates the network’s capacity to reroute flux in response to perturbations being critically diminished. We hypothesized that this rigidification manifests as shrinking flux-variability analysis (FVA) widths in key metabolic modules as the bioprocess approaches failure. By quantifying this loss of flexibility,^12^ we can identify active-constraint set shifts as tipping points signaling a transition between distinct metabolic regimes.

To convert these theoretical insights into practical diagnostics, we employed a dual-stage analysis. First, we utilized a genome-informed digital twin together with Latin-hypercube sampling (LHS) of acetate, oxygen, ammonium, and phosphate uptake bounds to systematically explore the intervention space and generate up to 2,000 metabolic-state contexts. For each context, we defined a metabolic regime *a priori* by identifying which uptake-bound exchange has the most-binding shadow price (e.g., O_2_-limited, N-limited, Ac-limited), yielding mutually exclusive condition labels. Within this space, we extracted interpretable flexibility features by computing targeted FVA on a curated panel of central-carbon, respiratory, acetate-uptake, and N-biosynthesis reactions: For each target reaction, FVA returns the minimum and maximum flux values consistent with retaining 95% of the optimal biomass flux, and the interval width (v_max_–v_m_ᵢ_n_) is recorded as a per-reaction “metabolic degree of freedom” feature that serves as a proxy for the cell’s remaining adaptive capacity.^12^

Second, we trained XGBoost^13^ learners on the resulting width vector to predict (1) the regime label (classification) and (2) a normalized growth-potential index defined as the per-condition objective value divided by the dataset-level maximum (regression), and applied SHapley Additive exPlanations (SHAP)^14^ attribution to identify which reactions’ flexibility losses are driving each prediction; PC-algorithm-based causal-structure discovery, evaluated with bootstrap stability over the SHAP-attributed reactions, then organizes the recurring co-collapses of degrees of freedom into a hypothesized rigidification map across the metabolic network.^15^^,^^16^^,^^17^ The pipeline therefore differs from point-flux-based diagnostics by diagnosing the collapse of metabolic flexibility rather than estimating a single optimal flux state.

We demonstrated the utility of this platform using aerobic growth of *Stenotrophomonas maltophilia* SO-1 (a non-model microorganism of growing industrial interest) on acetate as a controlled testbed. By anchoring the digital twin with sparse, standardized harvest-time readouts and computationally expanding the intervention space, we successfully recovered regime boundaries and identified the specific metabolic modules responsible for limiting performance. This study provides a proof-of-concept for a new class of intervention-aware digital twins that can generate testable mechanistic hypotheses and prioritize troubleshooting targets, even in systems where biological knowledge is limited and real-time observability is problematic.

## Results

### The intervention-aware digital twin produces regime maps, flexibility-collapse signatures, and rigidification hypotheses

**Figure 1:**
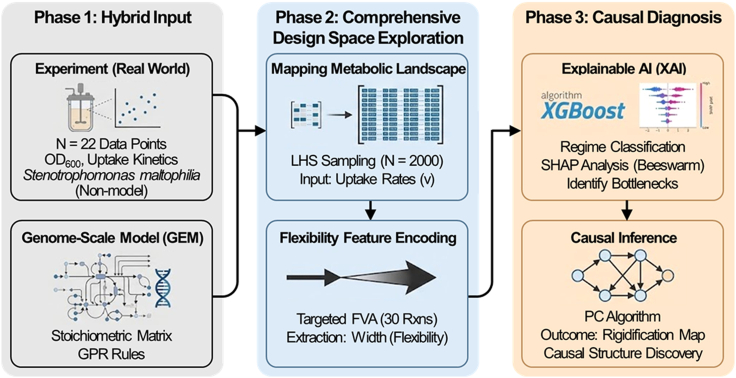
A schematic overview of the intervention-aware diagnostic platform (A) Model anchoring using experimental growth data. (B) Design-space exploration via LHS of the uptake constraints. (C) The diagnostic workflow combines metabolic flexibility encoding, SHAP-based explainability, and causal-structure discovery.

We developed an intervention-aware diagnostic platform that converts sparse experimental anchors into operationally interpretable outputs (Figure 1). The workflow yields three complementary diagnostic artifacts: (1) a regime map defined by the identity of the active limiting constraint, (2) flexibility-based signatures derived from targeted FVA widths that localize bottleneck proximity, and (3) an inferred rigidification map that organizes bottleneck hypotheses into an upstream-to-downstream cascade suitable for intervention prioritization.

### Standardized 32-h harvest-time anchors constrain the genome-informed digital twin across the nutrient and stress axes

**Figure 2:**
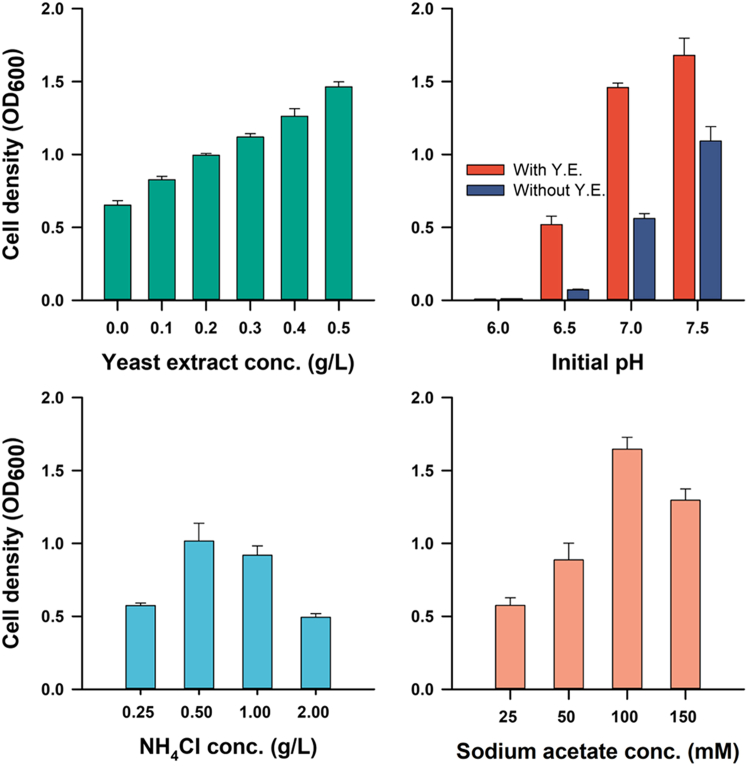
Experimental anchor conditions used to constrain the digital twin for *S. maltophilia* SO-1 acetate-based cultivation Endpoint growth (OD_600_, 32 h) measured across (A) yeast extract (YE; 0–0.5 g/L), (B) initial pH (6.0–7.5) with and without YE, (C) NH_4_Cl (0.25–2.0 g/L; YE fixed at 0.2 g/L to minimize background nitrogen), and (D) sodium acetate (25–150 mM) in phosphate-buffered acetate medium (30 °C, 200 rpm). The error bars denote mean ± SD (*n* = 3).

To ground the digital twin in experimentally observed physiology under sparsely observable conditions, we assembled a deliberately limited anchor dataset consisting of endpoint OD_600_ measurements at a standardized harvest time (32 h; *n* = 22) in phosphate-buffered acetate minimal medium (Figure 2). This fixed-time readout provides a consistent, decision-relevant phenotype for anchoring across experimental sets while not assuming substrate depletion under every set of conditions.

A GEM for *S*. *maltophilia* SO-1 was reconstructed using CarveMe^18^ from whole-genome sequencing to provide the stoichiometric and gene-protein-reaction backbone for the digital twin (Figure S1). The curated Escher map^19^ in Figure S2 summarizes the core network context used for targeted feature extraction and interpretation.

Across four perturbation sweeps, the anchors revealed interpretable saturation and inhibition patterns that serve as guardrails for plausible growth responses. Yeast extract (YE) supplementation increased endpoint OD_600_ monotonically over the tested range (0–0.5 g/L; Figure 2A). Initial pH strongly modulated growth and showed a clear interaction with YE: Without YE, growth remained near zero at acidic pH and increased toward neutral/alkaline conditions, whereas YE supplementation shifted the response upward across the same pH range (Figure 2B). The ammonium chloride (NH_4_Cl) titration exhibited a non-monotonic response, with improved growth at intermediate NH_4_Cl levels and reduced growth at the highest concentration tested (Figure 2C); to isolate inorganic nitrogen availability from confounding complex nutrients, this titration was conducted with YE fixed at 0.2 g/L (Figure 2C). Finally, the acetate concentration sweep showed an optimum at intermediate acetate levels, followed by decreased growth at the highest acetate concentration tested (Figure 2D).

Together, these results provide a controlled substrate-stress axis that could be used later to sharpen regime boundary interpretation, while these standardized harvest-time anchors establish a minimal yet structured experimental reference that constrains the digital twin to plausible behavior before large-scale intervention-labeled exploration.

### Intervention-labeled design-space exploration reveals regime structure and discontinuous boundaries via active-constraint switching

We expanded the sparse anchors to generate an intervention-labeled design space through LHS of uptake-constraint vectors (*n* = 2,000; Figure 1). Each simulated condition was labeled by its active limiting constraint using dual information (shadow prices), thereby making regime identity a mechanistically grounded label and enabling diagnosis as a question of constraint identity rather than outcome magnitude only.

**Figure 3:**
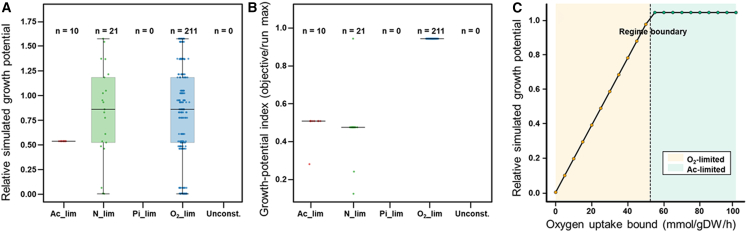
Regime-level variation in simulated growth potential and oxygen-dependent regime transition (A) Distribution of relative simulated growth potential across analyzed conditions grouped by the active limiting constraint identified via shadow prices. Numbers above each group indicate the number of conditions in that regime class. (B) Distribution of the normalized growth-potential index (objective/run max) across the same regime classes. (C) Relative simulated growth potential across a targeted oxygen uptake sweep (0–100 mmol gDW^−1^ h^−1^), showing the transition from O_2_-limited to acetate-limited regimes at the regime boundary.

Across the sampled design space, the distribution of simulated growth potential differed substantially by regime (Figure 3A), indicating that growth is not simply a single continuous landscape but rather a regime-conditioned behavior. Within the sampled uptake ranges, oxygen-limited (*n* = 211) occupied the largest proportion of feasible conditions, while nitrogen-limited (*n* = 21) and acetate-limited (*n* = 10) were less frequent. Meanwhile, limited phosphate availability and an unconstrained regime were not observed in this space (Figure 3A). To connect discrete regime identity to a continuous notion of feasible growth capacity, we also computed a normalized growth-potential index (objective/run-max) across the sampled conditions (Figure 3B). Because this index is scaled by the maximum objective value within the same simulation run, values closer to 1 indicate higher feasible growth potential, whereas values closer to 0 indicate stronger growth limitation. This allowed us to jointly analyze regime switching and within-regime changes in feasible growth capacity.

### A targeted oxygen sweep exposes a sharp transition from oxygen-limited to acetate-limited growth

Because limited oxygen availability dominated the feasible space, we performed a targeted oxygen uptake sweep while holding other constraints fixed. Simulated growth potential appeared to increase linearly with oxygen uptake capacity until reaching a distinct regime boundary, after which growth plateaued and the active constraint switched from oxygen-limited to acetate-limited (Figure 3C). This oxygen-to-acetate switch illustrates a core premise of the platform: Underperformance is often governed by not only smooth degradation but also structural reorganization of the feasible metabolic space, which is observable as a discrete change in the active-constraint set. Operationally, this boundary provides a diagnostic interpretation that a single continuous predictor cannot; it distinguishes whether improving oxygen transfer will increase growth (the oxygen-limited side) or will no longer help because a different constraint (carbon uptake/acetate) has become functionally limiting (the acetate-limited side).

### Flexibility features enable explainable regime diagnosis under flux non-identifiability

A persistent obstacle to GEM-based diagnosis is flux non-identifiability: Many flux vectors can satisfy the same constraints, thereby making point flux values unstable as diagnostic targets. We therefore encoded intracellular state using metabolic flexibility rather than a single flux solution, computing targeted FVA widths (30 curated reactions/modules) as flexibility features (Figure 1). Narrow widths indicate reduced rerouting capacity (rigidification), which provides an interpretable marker for bottleneck proximity.

**Figure 4:**
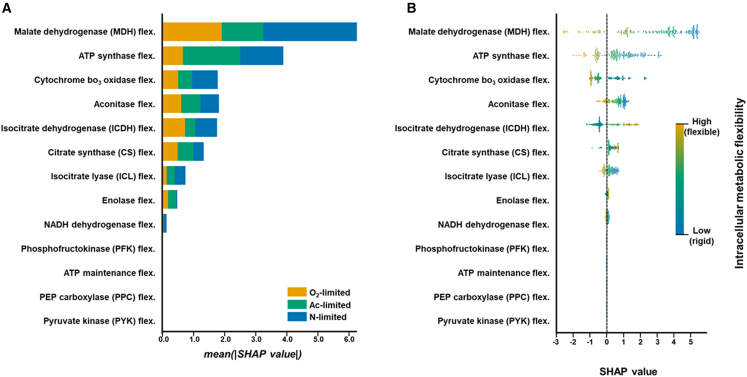
Identification of diagnostic flexibility features for metabolic regime classification (A) Regime-discriminating features: top-ranked flexibility features ranked by mean absolute SHAP values from the XGBoost classifier; stacked bars indicate the class-wise contribution to oxygen-, acetate-, and nitrogen-limited predictions. (B) An SHAP beeswarm summary for the oxygen-limited class showing how feature values (flexibility; high to low) drive the model output. Regime-specific SHAP summaries for the nitrogen- and acetate-limited classes are shown in Figure S3. For the global SHAP summaries in Figures 4 and 5, features were ranked from the extended ∼300-feature FVA-width universe described in the methods (Feature-panel ablation paragraph); the curated 30/42-reaction panel listed in Table S4 is retained as the mechanistic interpretability layer that supports the narrative in Figures 4, 5, and 6.

Using these flexibility features, we trained an explainable classifier (XGBoost) to predict regime identity and used SHAP attribution to identify which flexibilities carry diagnostic information (Figure 4). The global ranking process concentrated regime-discriminating signal in a small subset of features dominated by core energy and central carbon metabolism—tricarboxylic acid (TCA)-cycle and glyoxylate-shunt flexibilities (e.g., malate dehydrogenase, aconitase, isocitrate dehydrogenase, citrate synthase, and isocitrate lyase) together with respiratory/adenosine triphosphate (ATP)-related modules (ATP synthase, ATP maintenance, cytochrome bo3 oxidase, and nicotinamide adenine dinucleotide (NADH) dehydrogenase) and supporting glycolytic/anaplerotic reactions (enolase, phosphofructokinase, pyruvate kinase, and phosphoenolpyruvate (PEP) carboxylase) (Figure 4A). The SHAP beeswarm summary in Figure 4B shows that regime classification is driven by structured patterns in flexibility: Systematic shifts in key module flexibilities contribute disproportionately to regime assignment. Furthermore, regime-specific SHAP summaries for the nitrogen- and acetate-limited contexts show mechanistically coherent signatures that are distinct from the oxygen-limited context (Figure S3).

### Flexibility collapse defines a continuous growth-potential axis and reveals non-linear interactions across modules

**Figure 5:**
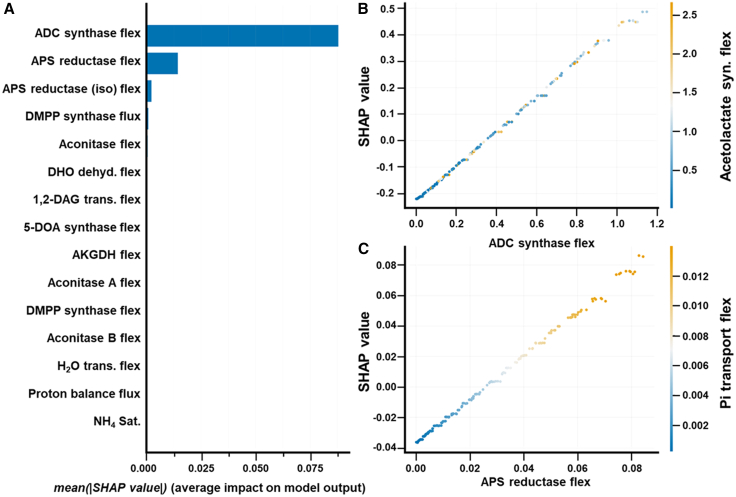
Determinants of growth-potential variation and non-linear tipping points (A) Global importance ranking of flexibility features derived from the XGBoost regression model for predicting the normalized growth-potential index. (B and C) SHAP dependence plots for ADC synthase and APS reductase, showing non-linear effects on the predicted growth-potential index. The non-linearity is quantitatively supported by piecewise-linear regression against a single-linear baseline (F-test p < 1e−3 for all top-3 severity features), with the ADCS breakpoint located at FVA width ≈0—a data-driven flexibility-collapse tipping point consistent with the rigidification interpretation in Figure 6.

Beyond regime identity, operational troubleshooting requires identifying what drives variation in feasible growth capacity. We therefore trained an explainable regression model to predict the normalized growth-potential index from flexibility features and ranked contributors using SHAP (Figure 5A). In contrast to regime classification, the regression ranking was dominated by a distinct subset of flexibilities, with particularly strong contributions from aminodeoxychorismate (ADC) synthase flexibility and adenosine phosphosulfate (APS) reductase flexibility, followed by additional biosynthetic and central metabolism-linked features (Figure 5A). This shift indicates that what distinguishes regimes is not necessarily identical to what governs variation in growth potential, thereby suggesting the need for both classification and regression views in a diagnostic workflow.

The SHAP-dependence plots in Figure 5B reveal structured, non-linear effects and interactions consistent with a flexibility-collapse interpretation. For example, the impact of ADC synthase flexibility on the predicted growth-potential index varies systematically and is modulated by acetolactate synthase flexibility, while the impact of APS reductase flexibility is modulated by phosphate transport flexibility (Figure 5C). Extended dependence plots for additional top-ranked features (ranks 3–6) show consistent non-linear patterns as flexibilities approach low-width states (Figure S4), implying that feasible growth potential can change disproportionately when key degrees of freedom collapse.

### A coordinated flexibility structure supports a bootstrap-stable-inferred rigidification map

Because bottlenecks can propagate across modules rather than acting in isolation, we examined pairwise relationships among the predominant flexibility features. The correlation heatmap in Figure S5 reveals strong covariation among multiple biosynthetic and transport flexibilities alongside structured anti-correlations with specific central metabolism flexibilities, which is consistent with coordinated rigidification rather than independent single-reaction failure.

**Figure 6:**
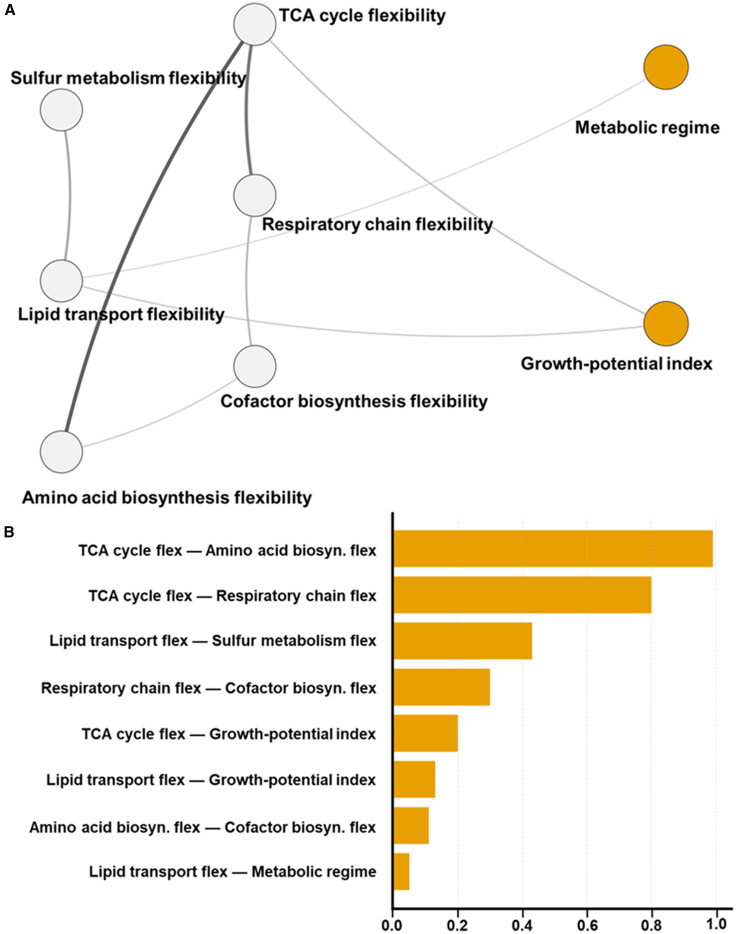
Causal rigidification map inferred by applying the PC algorithm (A) An undirected adjacency graph visualizes inferred dependencies between module-level flexibility variables and the normalized growth-potential index; edge thickness encodes bootstrap stability frequency. (B) Bootstrap stability scores for the inferred edges (100 iterations); edges are undirected because the bootstrap-stable equivalence class produced by the PC algorithm contained no oriented edges.

To organize diagnostic signatures into an intervention-relevant hierarchy, we applied causal-structure discovery via the Peter-Clark (PC) algorithm to variables spanning regime context, module-level flexibility features, and the normalized growth-potential index. This yielded an inferred rigidification map that organizes dependencies into a hierarchical structure (Figure 6A). Bootstrap resampling provides an internal robustness indicator, with a subset of edges persisting with high stability across iterations and forming a backbone of consistently inferred dependencies (Figure 6B). We interpreted this rigidification map not as causal proof, but as a hypothesis-prioritization structure that converts ranked features into an actionable sequence: What to evaluate first, what is likely to occur downstream, and which interventions are plausible first moves to restore flexibility.

Benchmarking the unchanged pipeline against simpler machine learning (ML) learners (logistic regression, random forest) and point-flux baselines (pFBA, FVA midpoint, flux balance analysis (FBA) objective) on the same curated panel and 5-fold cross-validation split (Figures S7 and S8) confirmed that the diagnostic signal is carried by the flexibility-interval representation rather than by a uniquely optimal learner. Most critically, the FVA-width representation outperformed the strongest pFBA point-flux baseline by Δ macro-F1 = +0.066 (0.972 vs. 0.906) on cross-organism transfer to *E. coli* iML1515 (*n* = 244 conditions).

### Prospective validation closes the diagnostic loop and flags non-stoichiometric constraints

**Figure 7:**
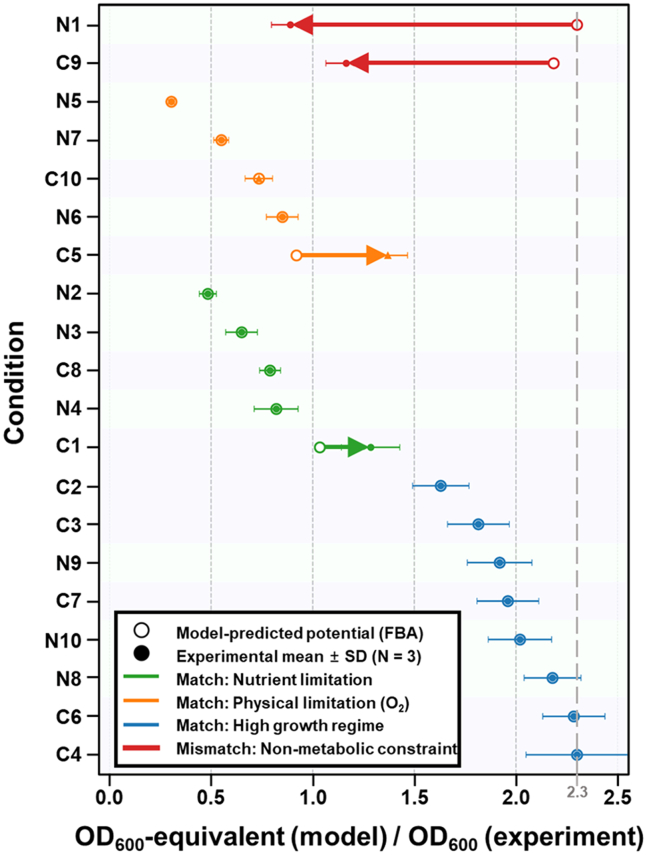
Prospective validation and diagnostic interpretation using the model-experiment comparisons Comparison of model-predicted growth potential (FBA, open circles) against experimentally measured endpoint OD_600_ values (filled circles; mean ± SD, *n* = 3) across the validation dataset (Table S3). The comparisons are categorized by diagnostic regime: (green) Nutrient-limited conditions show stoichiometric agreement, (orange) physical limitation (O_2_ transfer) conditions where the model accounts for restricted aeration, (blue) high-growth conditions showing consistent metabolic potential, and (red) mismatch regimes in which the model predicted high potential whereas the experimental outcomes showed suppression. We interpreted these gaps as diagnostic signals identifying non-stoichiometric bottlenecks (e.g., toxicity) not encoded in the metabolic network.

Finally, we evaluated whether the diagnostic framework could be generalized to the designed perturbations by conducting prospective experiments involving (1) limited-nutrient separation, (2) acetate-concentration sweeps, and (3) reduced oxygen-transfer contexts (mid-O_2_ and strict low-O_2_ setups, as defined in Table S3; condition sets C and N). We compared experimental endpoint growth (mean ± standard deviation (SD), *n* = 3) against model-predicted growth potential (biomass objective; FBA) and interpreted agreement/mismatch in the context of regime diagnosis (Figure 7).

Across many sets of conditions, the observed growth aligned with the model’s regime-level expectations, with distinct agreement patterns for nutrient-limited, oxygen-transfer-limited, and high-growth regimes (Figure 7). The validation also highlighted contexts where large model-experiment gaps emerged (Figure 7, red). Rather than treating these solely as model failures, we interpreted them as diagnostic signals pointing to non-stoichiometric constraints not encoded in the stoichiometric twin (e.g., physicochemical inhibition, regulation, and/or additional stress processes). In this sense, prospective validation closes the diagnostic loop: The platform can (1) classify limitation regimes when metabolic constraints predominate and (2) identify when additional non-stoichiometric mechanisms likely control underperformance, thereby prioritizing what should be measured or modeled next. A quantitative rank-ordering of the absolute model-experiment residuals across the C-series and N-series identifies C9 (140 mM acetate, high-O_2_) and N1 (154 mM acetate, high-O_2_) as the largest mismatches (Figure 7, red); in both cases the FBA model over-predicts growth potential while the measured OD_600_ is substantially suppressed. Both conditions sit at very high acetate concentrations, and the mismatch direction is consistent with non-stoichiometric inhibition (e.g., acetate-induced weak-acid stress) that the stoichiometric twin does not encode. Adding more flexibility features cannot lower the predicted growth ceiling, so the red mismatches are interpreted as a diagnostic signal for non-stoichiometric biology rather than as feature insufficiency.

## Discussion

### From simulation to diagnosis: Why an intervention-aware digital twin is useful?

This work establishes an intervention-aware digital-twin diagnostic platform that converts sparse experimental anchors into mechanistically interpretable diagnostic artifacts. The central shift is to treat deliberate perturbations as interventions and to use a genome-informed digital twin not merely as a predictor but also as a generator of an intervention-labeled design space in which regime boundaries and bottleneck signatures can be learned and interpreted. In practical terms, the platform was designed to answer the questions that arise when cultures underperform in practice: Which constraint is actively limiting? Where is flexibility collapsing? Which bottleneck cascade is most consistent with the observed context? What should be tested next?

A core advantage of this strategy is that it yields regime-aware diagnosis. For many process deviations, learning to smoothly map inputs in terms of growth can obscure the fact that the identity of the limiting constraint can change abruptly. By labeling regimes through the active-constraint set, the platform explicitly captures discontinuous boundaries, such as the oxygen-to-acetate switch observed in the targeted oxygen sweep, and provides a more actionable diagnostic context than a single continuous predictor. This distinction matters operationally because it separates conditions where increasing oxygen transfer should improve performance from those where doing so is no longer helpful due to a different constraint having become functionally limiting.

Agreement between the model potential and experimental outcomes supports the interpretation that the digital twin can diagnose nutrient-limited regimes when stoichiometric constraints predominate, while systematic mismatches can help to identify contexts likely governed by non-stoichiometric mechanisms (e.g., inhibition, regulatory effects, and physicochemical stress) that are not encoded in the stoichiometric matrix. In this framework, rather than treating mismatches as simple model failures, the diagnosis prioritizes which additional mechanisms should be measured or modeled next.

### Why flexibility is a robust diagnostic representation under non-identifiability

Although GEMs encode mechanistic constraints, they do not uniquely determine intracellular flux states.^20^ This non-identifiability is often treated as a diagnostic limitation because point flux predictions can vary widely without changing growth. Our results support a practical alternative: Metabolic degrees of freedom quantified via targeted FVA widths can be used to summarize the structure of the feasible space^12^ and represent the system’s metabolic buffering capacity. In this representation, a high number of degrees of freedom implies that the network retains multiple rerouting options to buffer against perturbations. Conversely, bottleneck proximity becomes interpretable as a collapse of these degrees of freedom; i.e., the exhaustion of buffering capacity. The observed SHAP-dependence patterns are consistent with this buffering-collapse view of impairment: Once the key degrees of freedom approach zero, the network becomes metabolically brittle, and feasible growth potential changes disproportionately.

Flexibility features also offer a portable diagnostic abstraction for non-model microbial culture systems. They can be defined at different granularities (i.e., individual reactions, curated modules, subsystems), and the feature set can evolve as the reconstruction is curated without changing the underlying logic of diagnosis via collapse. This continuity is valuable in settings where model refinement is iterative and where measurement availability varies across laboratories or process scales. In other words, the same diagnostic workflow can remain stable even as the underlying mechanistic model becomes more complete.

### Beyond importance ranking: Rigidification maps as structured troubleshooting hypotheses

Although explainable learning provides scalable bottleneck prioritization, operational decision-making typically needs more than a ranked list. Troubleshooting requires a sequence: what to check first, what is likely to occur downstream, and what intervention is most plausible given the current metabolic regime. Causal-structure discovery supplies this missing layer by proposing a propagation structure that links upstream rigidification to downstream limitations and reduced growth potential. We therefore present the rigidification map not as an indicator of definitive physical causality, but as a structured diagnostic hypothesis map conditioned on the digital twin and the intervention-labeled dataset.

The bootstrap-stable backbone provides an indicator of internal robustness that is directly compatible with practical workflows. Edges that persist across resampling are natural candidates for high-priority follow-up validation, while unstable edges can be treated as lower-confidence hypotheses. This framing process aligns with how mechanistic troubleshooting is conducted in practice: The goal is not to prove causality in a single step, but to reduce the search space and prioritize the next experiments or process adjustments. Importantly, because the map is inferred from intervention-labeled contexts, it is more directly aligned with intervention decisions than structures learned from observational correlations alone.

### Practical workflow: What the platform returns and how it can be used

From an end-user perspective, the platform returns three complementary artifacts that map naturally onto decision-relevant questions:

1. A regime map (active-constraint identity): This clarifies whether performance is limited by oxygen transfer capacity, carbon uptake, nitrogen availability, and/or other constraints, and it identifies boundaries where the limitations switch. Such boundaries distinguish when adjusting a knob should help versus when it is no longer the controlling factor.
2. Flexibility-collapse signatures (SHAP-ranked FVA widths): These indicate which reactions or modules are losing rerouting capacity and provide interpretable signatures that can be monitored or targeted. Because collapse is defined in the feasible-space structure rather than a single flux estimate, it remains meaningful under flux non-identifiability.
3. A rigidification map (a bootstrap-stable dependency structure): This organizes bottleneck hypotheses into a dependency-informed set of follow-up priorities (e.g., restoring central-carbon degrees of freedom before targeting downstream biosynthesis), thereby guiding intervention prioritization without implying a fully oriented causal order. It provides a structured plan for follow-up validation and iterative model refinement.

The anchoring strategy also reflects a practical design constraint: For many non-model microbial culture settings, only limited measurements are available within decision windows. Standardized harvest-time growth anchors provide a minimal demonstration of feasibility under sparse observability. In more instrumented settings, additional in-process signals (e.g., off-gas, feed history, dissolved oxygen trajectory, capacitance) can be incorporated as constraints, labels, and/or auxiliary targets, thereby improving diagnostic resolution without changing the diagnostic logic. In this sense, the architecture is designed to scale from optical density-only baselines to richer measurement regimes.

### External *in silico* generalizability

To test whether the diagnostic logic transfers beyond *S. maltophilia* SO-1, we applied the unchanged pipeline to the public *E*. *coli* GEM iML1515 (*n* = 244 LHS conditions; 45-reaction curated panel). The pipeline reached macro-F1 = 0.972 and severity R^2^ = 0.978 without retuning, and the cross-organism representation benchmark on the same panel (Figure S8) shows that the FVA-width representation outperforms the strongest pFBA point-flux baseline by Δ macro-F1 = +0.066 on iML1515. The top SHAP features in both organisms map to functionally analogous central-carbon, respiratory, acetate-uptake, and N-biosynthesis modules. The framework therefore generalizes in formulation to a well-characterized model organism in silico; wet-lab confirmation in a second organism is acknowledged as an important next step (Limitations).

### Limitations of the study

This study deliberately emphasizes diagnosis under sparse anchoring conditions, which gives rise to several limitations when defining the scope of interpretation. First, the anchors are standardized harvest-time growth readouts rather than full-term surveillance metrics. While this choice preserves productivity-relevant underperformance signals under minimal observability, prospective evaluation with in-process streams will become important for real-time deployment and dynamic diagnosis. Second, digital-twin diagnosis depends on modeling assumptions (e.g., biomass formulation, maintenance, objective choice), albeit anchoring and robustness indicators help mitigate sensitivity. Third, causal-structure discovery infers statistical structure conditioned on the simulated intervention-labeled dataset and cannot be used on its own to establish physical causality; our recommended interpretation is hypothesis prioritization rather than causal proof.

These limitations are not unique to this system, and they motivate a clear next step: closing the loop by testing a small number of high-priority interventions suggested by the rigidification map, particularly in regimes where validation indicates consistent stoichiometric behavior versus those in which systematic mismatches suggest non-stoichiometric constraints. This iterative cycle, comprising anchors, exploring interventions, diagnosing via flexibility, prioritizing hypotheses, and validating, defines a practical route for hypothesis-prioritized diagnostic support in non-model microbial culture systems. We present the framework here as a proof-of-concept diagnostic platform supported by anchored experimental data *in S. maltophilia* SO-1 and *in silico* cross-organism generalization on *E. coli* iML1515; we do not claim a fully validated, deployable system, and full wet-lab confirmation in a second organism is acknowledged as an important next step.

We developed a digital-twin-based platform to assess potential bioprocessing problems from outcome prediction to diagnosis by explicitly treating perturbations as interventions and by interpreting the culture state through regime identity and metabolic degrees of freedom. By anchoring a genome-scale twin with minimal standardized harvest-time growth phenotypes, we showed that intervention-labeled exploration can reveal discontinuous regime boundaries and that targeted flexibility features (FVA widths) provide a robust, interpretable representation under flux non-identifiability. Explainable learning was used to convert these features into bottleneck-ranked diagnostic signatures, while causal-structure discovery was employed to organize them into an inferred rigidification map that supports hypothesis prioritization and structured troubleshooting rather than single-feature correlations. Experimental outcomes aligned with the platform correctly identifying the predominant metabolic limitation regime, while systematic gaps flagged likely non-stoichiometric constraints that should be targeted by follow-up measurements or model extensions. More broadly, the architecture was designed to remain consistent with an increase in measurement richness by incorporating in-process streams and/or molecular readouts without changing the core diagnostic logic. Future work will focus on iterative loop closure by testing a small set of map-prioritized interventions and extending the framework to additional non-model microorganism and bioprocess contexts where sparse observability is the norm.

## Resource availability

### Lead contact

Further information and requests for resources and reagents should be directed to and will be fulfilled by the lead contact, Changman Kim (cmkim@jnu.ac.kr).

### Materials availability

*Stenotrophomonas maltophilia* SO-1 strain isolated in this study is available from the lead contact upon request. Ethics approval was not required because this study used only a bacterial isolate and involved no human participants or animal subjects.

### Data and code availability

The assembled genome sequence of *S. maltophilia* SO-1 has been deposited at NCBI: GCF_052922585.1. The reconstructed genome-scale metabolic model (iSO1_933), original code, training data, and raw experimental validation datasets have been deposited at https://github.com/cmkim0408/Causal-AI-FBA and are publicly available as of the date of publication.

Any additional information required to reanalyze the data reported in this paper is available from the lead contact upon request.

## Acknowledgments

This work was financially supported by the Basic Science Research Program through the 10.13039/501100003725National Research Foundation of Korea (NRF), funded by the Ministry of Education (RS-2025-25438814). Additional support for the revision-stage computational analyses and postdoctoral research activities was provided by the Regional Innovation System and Education (RISE) Glocal University 30 program through the Gwangju RISE Center, funded by the Ministry of Education (MOE) and the Gwangju Metropolitan City, Republic of Korea (2026-RISE(Glocal University 30)-05-011).

## Author contributions

Conceptualization: C.K.; methodology: C.K. and H.C. (for genome analysis); software: C.K.; validation: C.K. and D.K.; formal analysis: C.K. and H.C.; investigation: C.K., D.K., and H.C.; data curation: C.K. and H.C.; writing—original draft: C.K.; writing—review and editing: C.K., H.C., and D.K.; visualization: C.K.; supervision: C.K.; project administration: C.K.; funding acquisition: C.K.

## Declaration of interests

The authors declare no competing interests.

## Declaration of generative AI and AI-assisted technologies in the writing process

During the preparation of this work, the author(s) used ChatGPT-5.2 in order to enhance the readability and language quality. After using this tool/service, the author(s) reviewed and edited the content as needed and take(s) full responsibility for the content of the published article.

## STAR★Methods

### Key resources table

**Table:** 

REAGENT or RESOURCE	SOURCE	IDENTIFIER
**Bacterial and virus strains**		

*Stenotrophomonas maltophilia* SO-1	This paper	

**Deposited data**		

Assembled genome sequence of *S. maltophilia* SO-1	This paper	NCBI: GCF_052922585.1
Reconstructed genome-scale metabolic model (iSO1_933), code, and experimental datasets	This paper; https://github.com/cmkim0408/Causal-AI-FBA	N/A

**Software and algorithms**		

Python	Python Software Foundation; https://www.python.org/	v3.12.11
Trim Galore	Martin^21^; https://github.com/FelixKrueger/TrimGalore	v0.6.10
SPAdes	Bankevich et al.^22^; https://cab.spbu.ru/software/spades/	v4.2.0
RagTag	Alonge et al.^23^; https://github.com/malonge/RagTag	v2.1.0
Prokka	Seemann^24^; https://github.com/tseemann/prokka	v1.15.6
eggNOG-mapper	Cantalapiedra et al.^25^; http://eggnog-mapper.embl.de/	v2.1.13
Proksee	Grant et al.^26^; https://proksee.ca/	v1.0.0a6
CarveMe	Machado et al.^18^; https://github.com/cdanielmachado/carveme	v1.6.4
COBRApy	Ebrahim et al.^27^; https://github.com/opencobra/cobrapy	v0.31.1
XGBoost	Chen & Guestrin^13^; https://xgboost.ai/	v3.1.3
SHAP	Lundberg & Lee^14^; https://github.com/shap/shap	v0.49.1
Causal-learn	Zheng et al.^17^; https://github.com/py-why/causal-learn	v0.1.4.4
SciPy	Virtanen et al.^28^; https://scipy.org/	v1.17.0

### Experimental model and study participant details

#### Microbial strains

*S. maltophilia* SO-1 was isolated from aged homemade vinegar and maintained in glycerol at −80°C. Prior to liquid cultivation, the strain was revived on nutrient agar plates.

### Method details

#### Culture conditions and anchoring

For digital-twin anchoring, cultures were grown in a defined phosphate-buffered acetate minimal medium containing 100 mM potassium phosphate buffer, sodium acetate as the primary carbon source (25–150 mM), NH_4_Cl as the nitrogen source (0.25–2.0 g/L), and YE as indicated (0.0–0.5 g/L). During NH_4_Cl optimization, YE was fixed at 0.2 g/L to support minimal growth while isolating the effect of inorganic nitrogen availability. In 250 mL flasks at 30 °C, standard high-O_2_ cultures used a 50 mL volume at 200 rpm, whereas oxygen limitation was induced by reducing agitation to 80 rpm (mid-O_2_), combined with an increased 100 mL volume and punctured foil caps for strict low-O_2_ conditions.

Growth was quantified via endpoint OD_600_ measurements at a standardized harvest time of 32 h. The experimental anchor dataset comprised *n* = 22 conditions spanning four variable sweeps (YE supplementation, initial pH with/without YE, NH_4_Cl titration, and acetate concentration) (Table S1).

#### Genome sequencing and annotation

Genomic DNA was prepared using the TruSeq DNA Nano Prep Kit and sequenced on an Illumina NextSeq2000 platform. Raw reads were trimmed to remove adapters and low-quality bases using Trim Galore (v0.6.10).^21^ *De novo* genome assembly was performed using SPAdes (v4.2.0),^22^ followed by chromosome-level scaffolding with RagTag (v2.1.0)^23^ using the *S. maltophilia* reference genome (LT906480.1) as a guide. The final assembly comprised 4,479,228 bp (∼4.48 Mb) with 66.67% GC content and 4,386 predicted coding sequences (CDSs) (Table S2). Functional annotation was performed using Prokka (v1.15.6)^24^ and eggNOG-mapper (v2.1.13)^25^ based on the COG, GO, EC, KEGG, CAZy, and PFAM databases. Genome visualization was performed using Proksee.^26^

#### Metabolic model reconstruction

A GEM (denoted iSO1_933) was reconstructed from the annotated genome using CarveMe (v1.6.4) with a universal bacterial template.^18^ The initial reconstruction was manually curated to refine reaction directionality and transport mechanisms, and minimal reference-guided gap filling was applied to enable biomass production under the experimentally defined acetate/ammonium minimal-medium context. Final model statistics (genes, reactions by type, metabolites) are reported in Table S2.

#### Modeling and design-space exploration

Constraint-based simulations were performed using flux balance analysis (FBA)^20^ by maximizing the biomass objective under steady-state mass balance and capacity constraints, which is defined as

$$\max\;\mu \;s.t.\;S_{v}=0,l_{j}\leq v_{j}\leq u_{j}$$

where *S* is the stoichiometric matrix, *v* is the flux vector, and [l_j_, u_j_] are the flux bounds. Environmental conditions were encoded by modifying exchange bounds for oxygen, acetate, ammonium, and other relevant nutrients. All simulations were implemented using COBRApy with the default *GLPK* solver and a numerical tolerance of 10^−6^.^27^

To generate an intervention-labeled dataset beyond sparse anchors, uptake-constraint vectors were sampled using LHS (*n* = 2,000) across predefined physiological ranges for the selected uptake bounds. For each sampled condition, FBA was solved and growth potential (μ) and dual variables were recorded. Of the 2,000 LHS-sampled uptake-constraint contexts, 242 feasible conditions with valid regime labels and complete FVA-width records (oxygen-limited *n* = 211; nitrogen-limited *n* = 21; acetate-limited *n* = 10) were retained for the diagnostic learning, SHAP analysis, and benchmarking reported in subsequent sections; the remaining samples were excluded due to infeasibility or incomplete FVA campaign storage.

#### Regime labeling and flexibilities

Metabolic regime labels were assigned using shadow prices (dual variables) associated with the exchange-reaction uptake bounds. A positive shadow price indicates that relaxing a bound would increase the growth objective, thereby identifying a functional limitation under that condition. Regime identity was assigned to the exchange bound with the largest positive shadow price. When multiple bounds had comparable sensitivities within 10^−5^, the condition was labeled as co-limited.

To map the oxygen-to-acetate transition, oxygen uptake capacity was swept from 0 to 100 mmol gDW^−1^ h^−1^ while holding the other uptake constraints fixed at reference values (sodium acetate: 50 mM, NH_4_Cl: 1.0 g/L, and YE: 0.5 g/L).

#### Feature engineering via targeted FVA

To address flux non-identifiability, the intracellular state was encoded using metabolic degrees of freedom rather than point flux estimates.^29^ A curated panel of 30 reactions/modules was selected for mechanistic interpretability, covering central-carbon (TCA/glyoxylate/glycolysis) reactions, respiration/ATP modules, acetate uptake/activation, and N-biosynthesis (full panel in Table S4); the choice is validated by the feature-panel ablation reported below. FVA was performed for these 30 targeted reactions/modules under near-optimal growth (*μ* ≥ 0.95 *μ*_opt_). For each target reaction, the degrees of freedom width was computed as

$$W=\nu_{\max}-\nu_{\min}$$

where *ν*_*max*_ and *ν*_*min*_ are the maximal and minimal feasible flux values under the constraints for quantifying the remaining rerouting capacity under the imposed condition.

A continuous normalized growth-potential index was computed for each condition as follows:

$$G_{i}=\frac{{objective}_{i}}{{objective}_{\max,run}}$$

where *objective*_*i*_ denotes the biomass objective value for condition *i* and *objective*_*max*,*run*_ is the maximum theoretical growth rate observed within the same simulation run, yielding a unitless score in [0, 1]. Higher values indicate greater feasible growth potential, whereas lower values indicate stronger growth limitation.

To verify that the curated panel choice does not impair predictive performance, we performed a six-level feature-panel ablation comparing the curated 30-reaction panel against Top-K SHAP-ranked panels (K = 10, 20, 50), the full set of available FVA-width features, and ten random 30-feature controls (Figure S6). Because the originally deployed FVA-width feature set was alphabetically truncated at FACOAL161 (120 reactions), we first performed a supplementary targeted FVA campaign on 180 additional reactions across all 242 stored (campaign × run × condition) tuples, with per-row replay verified against the originally stored objective value (Δ ≤ 5 × 10^−2^ for all rows). This expanded the deployed feature set from 120 to ∼300 width features and re-introduced paper-named TCA/glyoxylate/respiration anchors (MDH, ICDHx, ICDHyr, ICL, MALS, PYK, PPC, NADH16pp, FUM, etc.) that had been absent from the truncated set. Within this extended feature set, performance was largely insensitive to panel size (Δ macro-F1 ≤ 0.04 across panels of 10–300 features; random 30-feature controls reached macro-F1 = 0.981 ± 0.009), confirming that the diagnostic signal is broadly distributed and that the curated panel is retained as the interpretability layer of the framework rather than as a performance-optimal subset.

#### Explainable AI and causal discovery

Gradient-Boosted (XGBoost) tree models were trained to map degrees of freedom features to (1) regime labels and (2) growth-potential index values.^13^ Hyperparameters were tuned using stratified 5-fold cross-validation; performance was evaluated by strictly out-of-fold predictions. On the extended feature set (∼300 FVA-width features), the XGBoost classifier reached macro-F1 = 0.991 with per-class F1 = {0.976, 1.000, 0.998} for {N-limited, Ac-limited, O2-limited} and balanced accuracy = 0.984, and the regressor reached RMSE = 0.034, MAE = 0.004, R^2^ = 0.906. Model interpretability was assessed using SHAP (TreeExplainer), and bottleneck candidates were ranked by using their mean absolute SHAP values.^14^

To infer a bootstrap-stable dependency structure among the bottlenecks without relying on feature importance, causal-structure discovery was performed by using the PC algorithm on a matrix combining regime/context variables, module-level degrees of freedom variables, and growth-potential index values.^16^^,^^30^ Conditional independence was assessed using Fisher’s z-test with *α* = 0.05. Structure stability was evaluated via bootstrap resampling (100 iterations), with edges only being retained in the final rigidification map if their bootstrap stability score exceeded *τ* = 0.90.

#### Baseline benchmarking

To evaluate whether the diagnostic signal depends on a specific feature representation or learner, we benchmarked the unchanged pipeline along two orthogonal axes on the same *n* = 242 dataset and 5-fold cross-validation split: feature set × learner (Figure S7) and feature representation—FVA width (ours) vs. FVA midpoint, parsimonious-FBA point flux, and FBA objective alone (Figure S8)—using identical preprocessing and hyperparameter-tuning protocols. The representation benchmark was additionally repeated on the public *Escherichia coli* GEM iML1515 (*n* = 244 LHS conditions; 45-reaction panel) to test cross-organism robustness.

#### Prospective validation experiments

Prospective validation experiments were performed under conditions designed to encompass distinct regimes and oxygen-transfer contexts (Condition Sets C and N in Table S3). Validation experiments included limited-nutrient separation, acetate-concentration sweeps, and oxygen-transfer restriction conditions (mid-O_2_ and strict low-O_2_ setups, as defined in Table S3).

### Quantification and statistical analysis

Quantification and statistical analyses were performed using *SciPy*/*Statsmodels* in Python.^28^^,^^31^ The association between the model-derived growth-potential index and experimental endpoint OD_600_ (32 h) across the anchor conditions was evaluated using Spearman’s rank correlation (*ρ* = 0.53, *p* = 0.04). All statistical tests were two-sided unless otherwise stated.

#### Runtime and computational resources

All analyses were performed on a single CPU core (Python 3.12.11; xgboost 3.1.3; cobra 0.31.1; scikit-learn 1.8.0; shap 0.49.1; GLPK solver) with peak memory below 420 MB. Per-stage wall-clock times were: LHS sampling ≈0.0003 s (500 conditions); FBA batch ≈2.07 s (50 conditions); targeted FVA ≈0.42 s (10 conditions × 30 reactions); XGBoost classifier + SHAP ≈0.29 s (*n* = 242); XGBoost regressor + SHAP ≈0.06 s; PC-bootstrap causal discovery ≈0.32 s (25 iterations; the final Figure 6 stability map used 100 bootstrap iterations, with this stage scaling approximately linearly in the number of iterations). The complete pipeline executes end-to-end in ≈3.2 s, making it feasible for real-time bioprocess troubleshooting at single-condition scale and overnight design-space exploration at LHS-batch scale.
