# Supplementary material for: Causal AI digital twin for bioprocess bottleneck diagnosis via metabolic flexibility and rigidification maps
Source: iScience. 2026 Jul 16;29(8):116820. doi: 10.1016/j.isci.2026.116820 (PMC13400960; doi:10.1016/j.isci.2026.116820)
Supplement: Document S1. Figures S1–S8, and Tables S1–S4 [file mmc1.pdf]

iScience, Volume *29*

Supplemental information

Causal AI digital twin for bioprocess bottleneck diagnosis via metabolic flexibility and rigidification maps

Changman Kim, Hyeongwoo Choi, and Dukwoo Kim

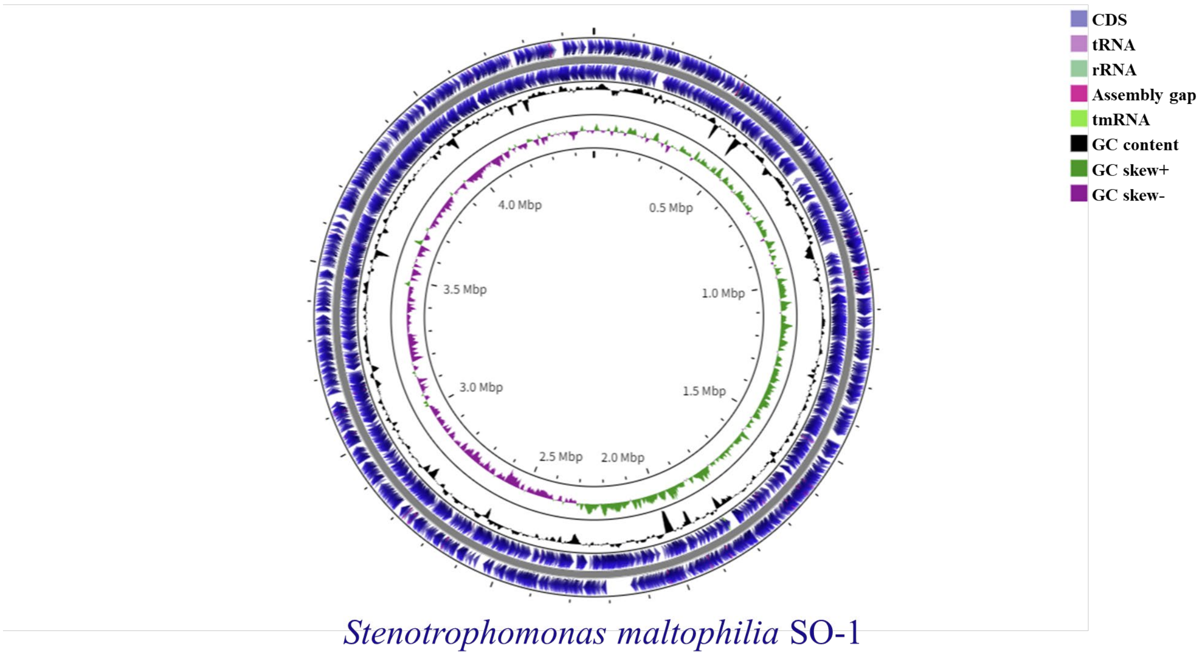

Figure S1. Circular genome map of *Stenotrophomonas maltophilia* SO-1

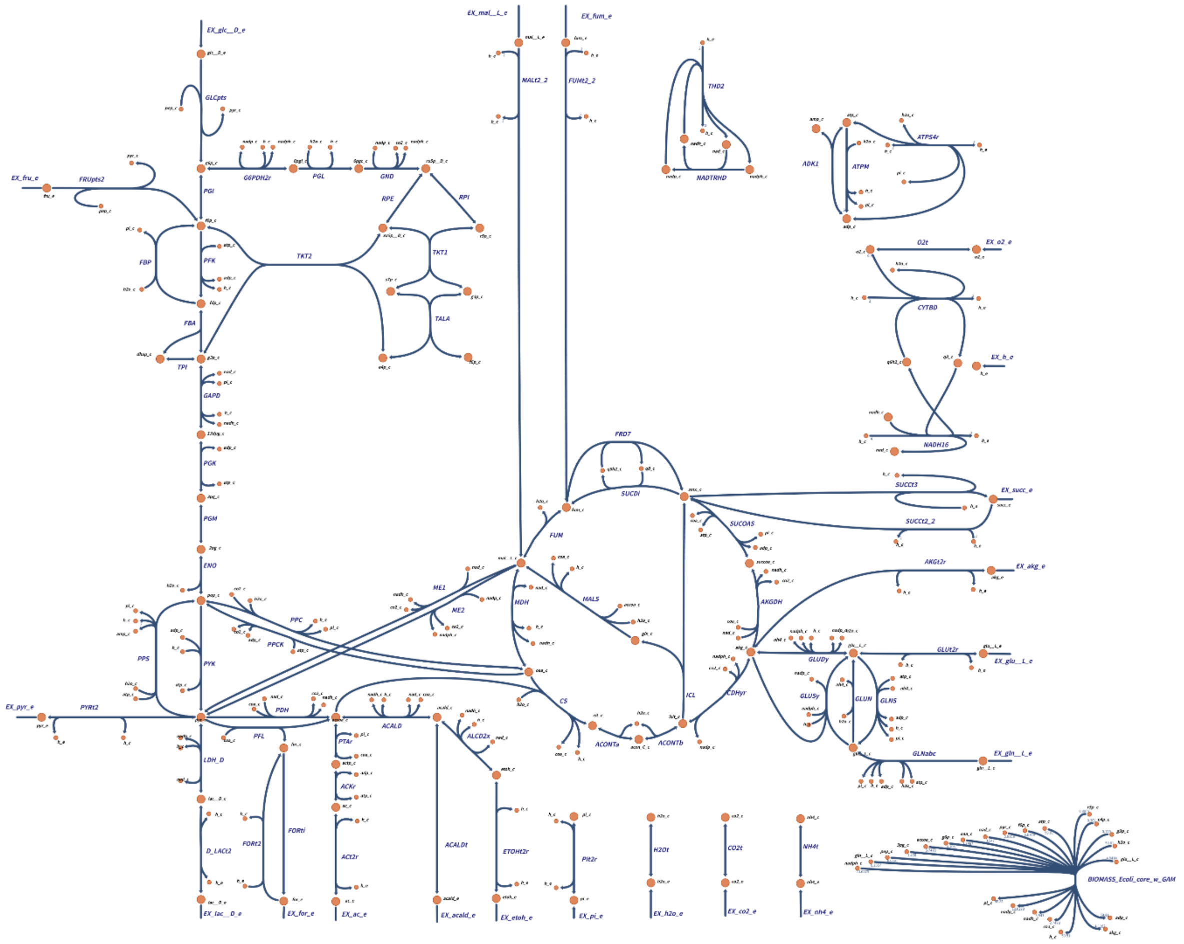

Figure S2. Curated Escher map of the core metabolic network used for interpretation and feature selection

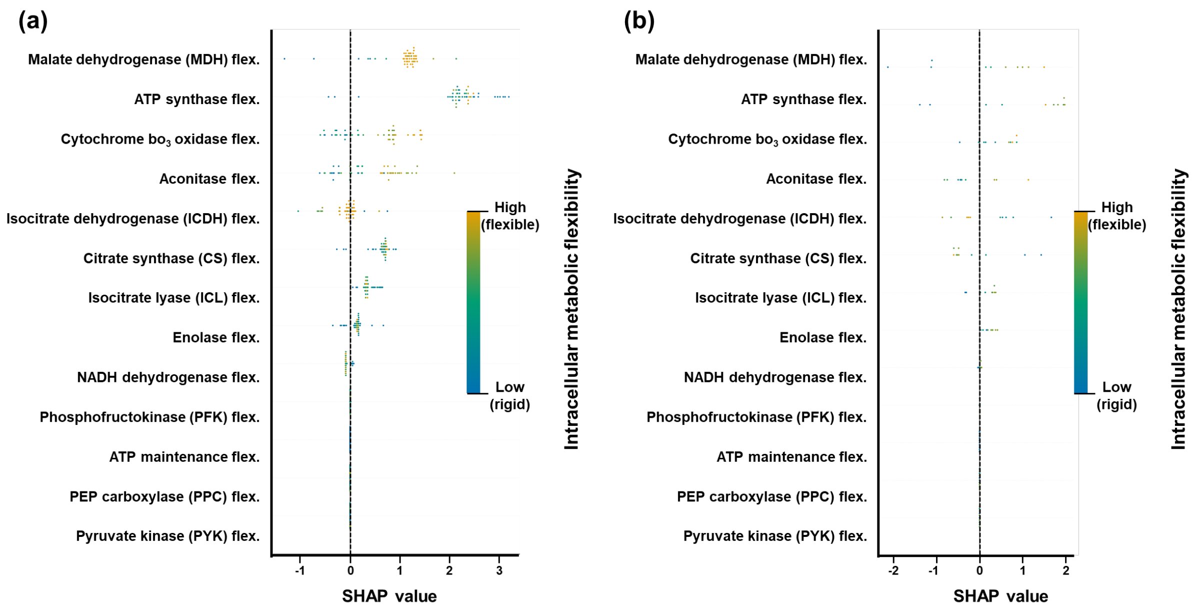

Figure S3. Regime-specific SHAP beeswarm summaries for the minority classes. (a) Nitrogen-limited class and (b) acetate-limited class. Each point represents a simulated condition; color indicates feature value (flexibility, high to low), and the dashed vertical line denotes zero contribution.

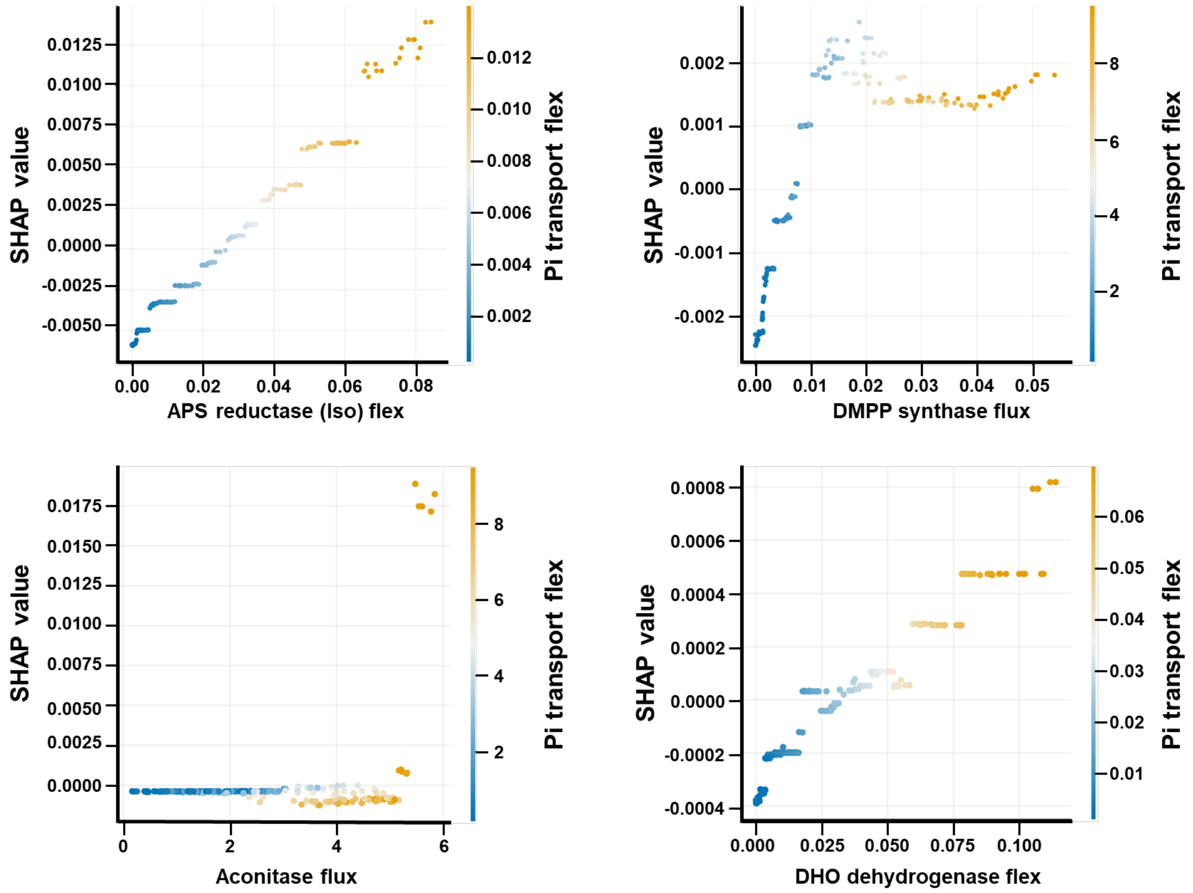

Figure S4. Extended SHAP dependence plots for additional top-ranked growth-potential drivers (ranks 3–6)

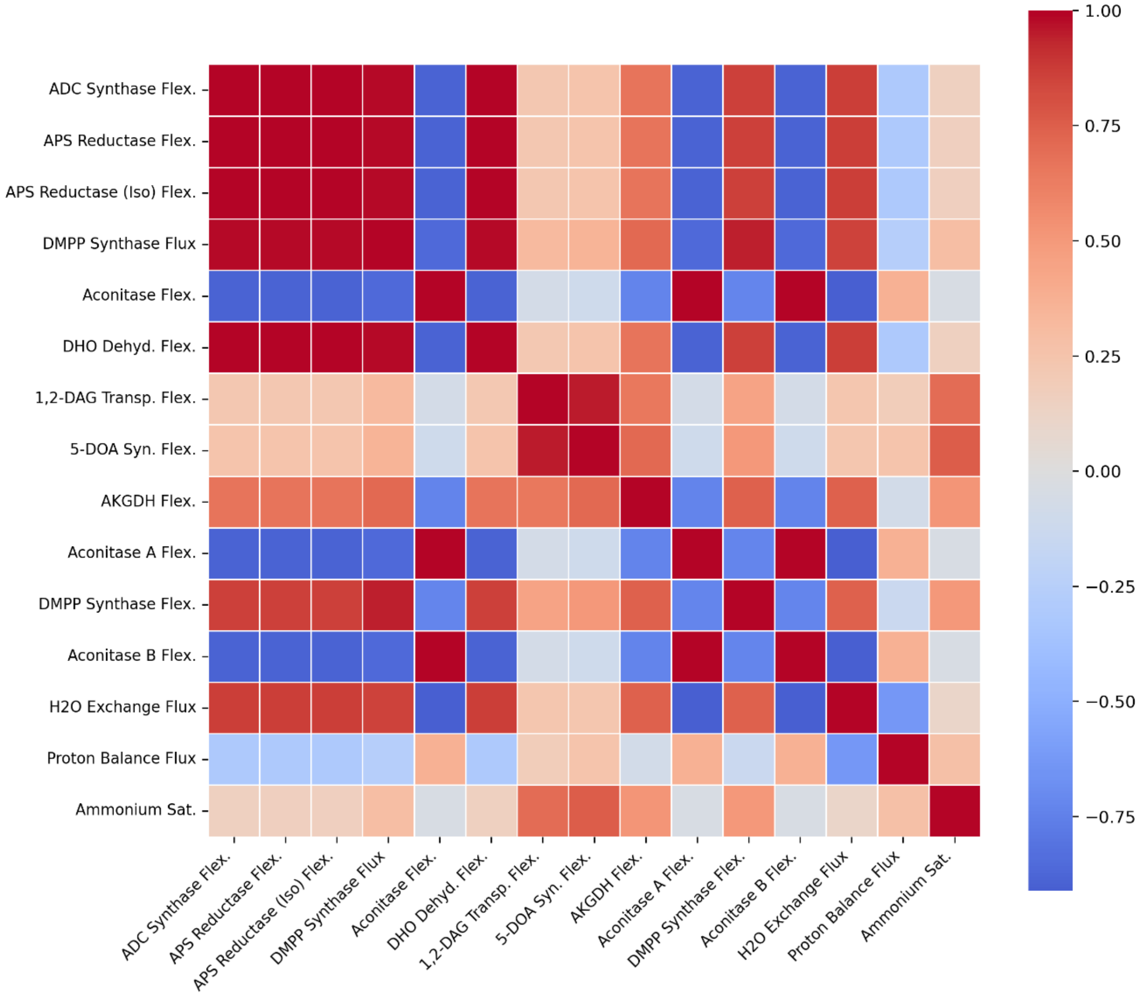

Figure S5. Correlation structure among predominant flexibility/flux signatures

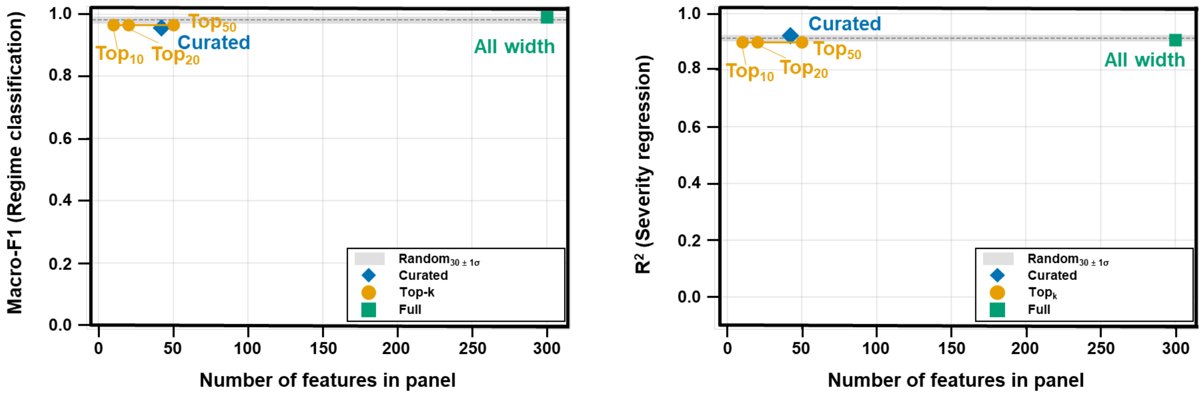

Figure S6. Feature-panel ablation on the diagnostic dataset (n = 242). Macro-F1 (regime classification, left) and severity R^2 (growth-potential regression, right) across six feature panels under identical 5-fold cross-validation: Top-K SHAP-ranked panels at K = 10, 20, 50 (orange), curated paper-aligned panel of n = 42 (blue), full ~300-feature superset (green), and ten random 30-feature controls (gray band, mean ± 1σ).

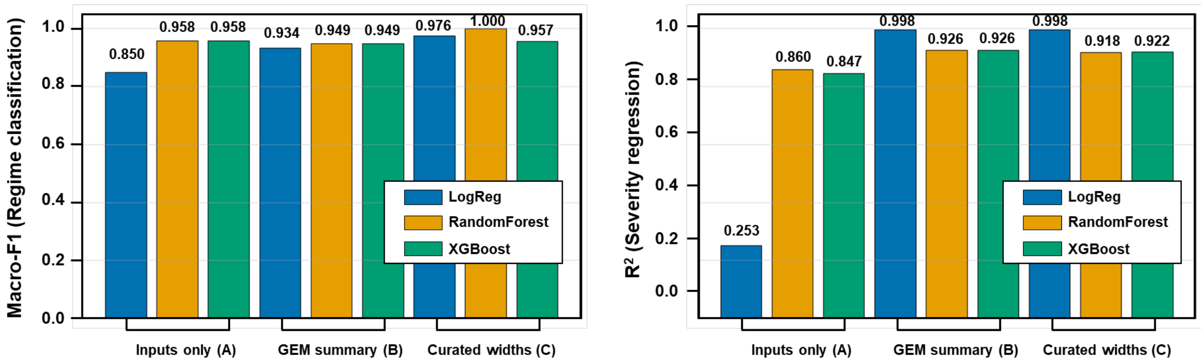

Figure S7. Feature-set × learner benchmark on the diagnostic dataset (n = 242, 5-fold cross-validation). Macro-F1 (regime classification, left) and R^2 (severity regression, right) across three feature sets — (A) uptake-bound inputs only (4 features), (B) FBA-objective plus per-anchor flux summary (9 features), (C) curated FVA-width panel (42 features) — each paired with logistic regression (blue), random forest (orange), and XGBoost (green). The high R^2 for panel B reflects target-construction overlap since severity = obj/obj_max.

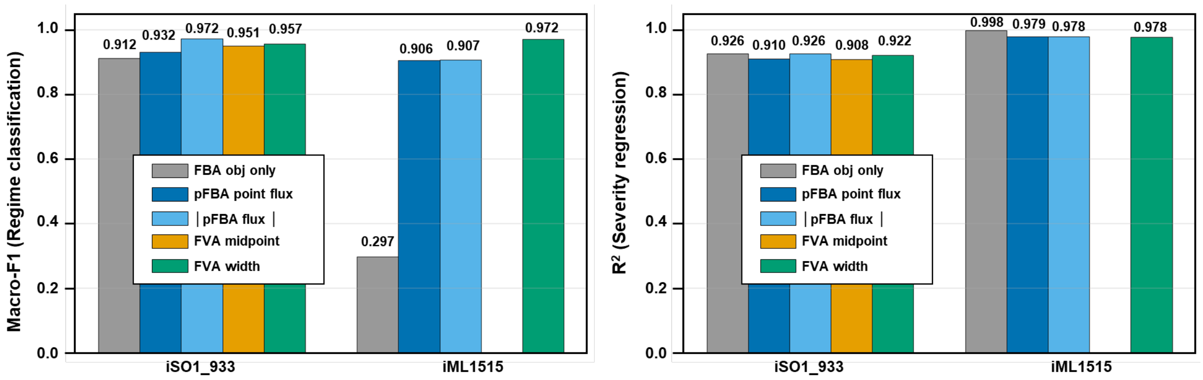

Figure S8. Point-flux vs flexibility-interval representation benchmark (same curated panel, same XGBoost, 5-fold cross-validation). Macro-F1 (left) and R^2 (right) across five feature representations — FBA objective only, signed pFBA flux, absolute pFBA flux, FVA midpoint, and FVA width (ours) — on iSO1_933 (*S. maltophilia* SO-1; n = 242, 42-reaction panel) and iML1515 (*E. coli*; n = 244, 45-reaction panel). On iML1515, FVA width outperforms the strongest pFBA baseline by Δ macro-F1 = +0.066 (0.972 vs 0.906). FVA midpoint was not computed for iML1515.

Table S1. Base medium composition, culture conditions, and parameter ranges for the 32-h harvest-time anchor experiments

Category	Parameter	Experimental Condition / Range
Base Medium	Source	*Stenotrophomonas maltophilia* SO-1 (from aged vinegar)
	Carbon Source	Sodium Acetate
	Nitrogen Source	NH_4Cl
	Supplements	Yeast Extract (YE)
	Buffer System	100 mM Potassium Phosphate
Culture Setup	Temperature	30°C
	Agitation	200 rpm
	Measurement	Endpoint OD_{600}
Variable Sweeps	Yeast Extract	0.0 – 0.5 g/L
	Initial pH	6.0 – 7.5
	NH_4Cl	0.25 – 2.00 g/L
	Sodium Acetate	25 – 150 mM

Table S2. Genome features of *Stenotrophomonas maltophilia* SO-1 and statistics of the reconstructed genome-scale metabolic model (iSO1_933)

Category	Feature	Statistic
Genome	Organism	*Stenotrophomonas maltophilia* SO-1
(Experimental)	Genome Size	4,479,228 bp (~4.48 Mb)
	GC Content	66.67%
	Total Coding Sequences (CDS)	4,386
Metabolic Model	Model Name	iSO1_933
(GEM)	Number of Genes	933 (Coverage: ~21.3%)
	Number of Reactions	2,092
	- Gene-associated	1,609
	- Transport	586
	- Exchange	219
	Number of Metabolites	1,367

Table S3. Prospective validation condition matrix (C-series and N-series) and diagnostic intent

Exp. ID	Design Source (Sampling Index)	Acetate (mM)	NH_4Cl (g/L)	YE (g/L)	Physical Condition (Aeration & Vessel)	Target Regime (Diagnostic Intent)
C-Series						
C1	Manual Selection	40	0.5	0.5	High O_2 (Standard)	Nutrient Limitation
C2	Manual Selection	80	1	1	High O_2 (Standard)	Nutrient Limitation
C3	Manual Selection	100	1	2	High O_2 (Standard)	Nutrient Limitation
C4	Manual Selection	100	2	5	High O_2 (Standard)	High Growth (Optimum)
C5	Manual Selection	100	1	1	Mid O_2 (50mL, 100rpm)	Physical Limitation (Aeration)
C6	Manual Selection	100	2	3	High O_2 (Standard)	High Growth
C7	Manual Selection	90	2	3	High O_2 (Standard)	High Growth
C8	Manual Selection	40	1	1	High O_2 (Standard)	Carbon Starvation
C9	Manual Selection	140	0.5	0.5	High O_2 (Standard)	Mismatch (Toxicity)
C10	Manual Selection	80	1	1	Mid O_2 (50mL, 100rpm)	Physical Limitation (Aeration)
N-Series						
N1	LHS Index #1834	154	1.4	1.2	High O_2 (Standard)	Mismatch (Predicted Safe)
N2	LHS Index #1615	55	0.4	0.2	High O_2 (Standard)	Nutrient Limitation
N3	LHS Index #645	68	0.8	0.4	High O_2 (Standard)	Nutrient Limitation
N4	LHS Index #892	75	0.9	0.5	High O_2 (Standard)	Nutrient Limitation
N5	LHS Index #1540	90	1	1	Strict Low O_2 (100mL, Foil)	Physical Limitation (Hypoxia)

N6	LHS Index #1734	85	1.2	1.1	Strict Low O_2 (100mL, Foil)	Physical Limitation (Hypoxia)
N7	LHS Index #429	88	1.1	0.9	Strict Low O_2 (100mL, Foil)	Physical Limitation (Hypoxia)
N8	LHS Index #1428	83	1.8	2.5	High O_2 (Standard)	High Growth (Sweet Spot)
N9	LHS Index #274	81	1.6	2.2	High O_2 (Standard)	High Growth
N10	LHS Index #277	99	1.9	2.8	High O_2 (Standard)	High Growth

Table S4. Curated 30/42-reaction panel for FVA-based flexibility features. The 30 reactions/modules listed in the main text expand to 42 width-features after including isoform variants (e.g., MDH/MDH2/MDH3, ACONT/ACONTa/ACONTb).

#	Reaction ID	Module / Function
1	EX_o2_e	Oxygen exchange (limiting)
2	EX_nh4_e	Ammonium exchange (limiting)
3	EX_pi_e	Phosphate exchange (limiting)
4	EX_co2_e	CO_2 exchange
5	EX_h_e	Proton exchange
6	EX_h2o_e	Water exchange
7	ATPS4rpp	ATP synthase (respiration / ATP)
8	ADK1	Adenylate kinase (energy)
9	AKGDH	α-Ketoglutarate dehydrogenase (TCA)
10	CYO1_KT	Cytochrome oxidase (respiration)
11	NADH16pp	NADH dehydrogenase (respiration)
12	CS	Citrate synthase (TCA)
13	ACONT	Aconitase (TCA)
14	ACONTa	Aconitase isoform a
15	ACONTb	Aconitase isoform b
16	ICDHyr	Isocitrate dehydrogenase (NADP, TCA)
17	ICDHx	Isocitrate dehydrogenase (NAD)
18	ICL	Isocitrate lyase (glyoxylate)
19	MALS	Malate synthase (glyoxylate)
20	MDH	Malate dehydrogenase (TCA)
21	MDH2	Malate dehydrogenase isoform 2
22	MDH3	Malate dehydrogenase isoform 3
23	FUM	Fumarase (TCA)
24	ACS	Acetyl-CoA synthetase (acetate activation)
25	ACSERL	Acetyl-CoA carboxylase / lipid
26	ENO	Enolase (glycolysis)
27	ENOPH	Enolase-phosphatase
28	PYK	Pyruvate kinase (glycolysis)
29	PYK3	Pyruvate kinase isoform 3

30	PPC	Phosphoenolpyruvate carboxylase (anaplerotic)
31	ACLS	Acetolactate synthase (BCAA)
32	ACLSa	Acetolactate synthase isoform a
33	ACLSb	Acetolactate synthase isoform b
34	ADCS	ADC synthase (severity SHAP top)
35	APSR	APS reductase (severity SHAP top)
36	APSR2	APS reductase isoform 2
37	GLNS	Glutamine synthetase (N biosynthesis)
38	GLUDy	Glutamate dehydrogenase (NADP)
39	ACGS	N-Acetylglutamate synthase (Arg pathway)
40	ARGSL	Argininosuccinate lyase
41	ARGSS	Argininosuccinate synthase
42	ASPTA	Aspartate aminotransferase
